# Investigating Whether Dissemination in Time Is Essential to Diagnose Relapsing Multiple Sclerosis

**DOI:** 10.1212/WNL.0000000000210274

**Published:** 2025-03-04

**Authors:** Wallace J. Brownlee, Michael A. Foster, Giuseppe Pontillo, Indran Davagnanam, Sara Collorone, Ferran Prados, Baris Kanber, Frederik Barkhof, Alan J. Thompson, Ahmed T. Toosy, Olga Ciccarelli

**Affiliations:** 1 Queen Square Multiple Sclerosis Centre, Department of Neuroinflammation, UCL Institute of Neurology, London, United Kingdom;; 2 NIHR University College London Hospitals Biomedical Research Centre, United Kingdom;; 3 Department of Radiology and Nuclear Medicine, Amsterdam UMC, Vrije Universiteit Amsterdam, the Netherlands;; 4 Department of Brain Repair & Rehabilitation, UCL Queen Square Institute of Neurology, Faculty of Brain Sciences, London, United Kingdom;; 5 Centre for Medical Imaging Computing, Department of Medical Physics and Biomedical Engineering, Faculty of Engineering Science, University College London, United Kingdom; and; 6 Universitat Oberta de Catalunya, Barcelona, Spain.

## Abstract

**Background and Objectives:**

The diagnosis of multiple sclerosis (MS) requires evidence of both dissemination in space (DIS) and time (DIT); oligoclonal bands (OCBs) in the CSF can substitute for DIT on MRI. We investigated whether DIT (or positive CSF) is necessary to make a diagnosis of MS in patients who fulfil a high number of DIS criteria.

**Methods:**

We prospectively recruited patients with a first demyelinating event evaluated with brain and spinal cord MRI within 3 months of onset. The patients were followed up clinically and with MRI. We retrospectively applied DIS criteria requiring lesions in ≥2/4, ≥3/4, or 4/4 regions typically affected in MS (periventricular, cortical/juxtacortical, infratentorial, spinal cord) and ≥2/5, ≥3/5, ≥4/5, and 5/5 regions (including the optic nerve) to baseline assessments. We investigated the performance of each set of DIS criteria for a diagnosis of MS using the 2017 McDonald criteria, requiring both DIS (lesions in ≥2/4 regions) plus DIT on MRI (gadolinium-enhancing and nonenhancing lesions, new T2 lesions at follow-up) or CSF-specific OCBs, as the gold standard.

**Results:**

We included 244 patients (mean age 32.5 years, 154 [63%] female); 187 (77%) patients were diagnosed with MS using the 2017 McDonald criteria over a mean follow-up of 11.2 years. DIS alone, requiring lesions in ≥2/4, ≥3/4, or 4/4 regions, exhibited reducing sensitivity (84%, 58%, and 26%, respectively) and increasing specificity (91%, 98%, 100%) for an MS diagnosis. In 112 (46%) patients with optic nerve assessment with orbital MRI or visual evoked potentials, DIS in ≥2/5, ≥3/5, ≥4/5, or 5/5 regions also resulted in reducing sensitivity (96%, 83%, 61%, 30%) and increasing specificity (44%, 83%, 100%, 100%) for MS diagnosis. We propose a diagnostic algorithm for MS in patients with a first demyelinating event based on the number of DIS regions fulfilled.

**Discussion:**

In patients with a first demyelinating event, DIS in ≥4 regions typically affected in MS is highly specific, indicating an extremely low risk of false-positive results, and misdiagnosis. Using DIS in ≥4 regions would reduce the need for follow-up MRI or CSF examination in all patients with suspected MS, streamlining the diagnostic process. Limitations include an over-representation of patients with optic neuritis at onset, a low rate of CSF examination, and lack of optical coherence tomography data.

## Introduction

The requirement for dissemination in time (DIT) and dissemination in space (DIS) has been the cornerstone of multiple sclerosis (MS) diagnostic criteria for nearly 60 years.^1,2^ MRI is routinely used to provide evidence for DIS and DIT,^2^ resulting in earlier diagnosis and treatment.^3,4^ Using the 2017 McDonald criteria, DIS on MRI requires ≥1 T2 lesion in 2 of 4 CNS regions typically affected in MS (periventricular, cortical/juxtacortical, infratentorial, spinal cord).^5^ It is anticipated that future iterations of the McDonald criteria will include the optic nerve as a fifth region, based on new evidence.^6-8^ A second attack, gadolinium-enhancing and nonenhancing lesions, or new T2 lesion(s) on MRI, all provide evidence for DIT.^5^ CSF-specific oligoclonal bands (OCBs) are an alternative to clinical or MRI evidence of DIT.^5,9^ Both DIS plus DIT (or CSF-specific OCBs) are required for a diagnosis of MS.^5^

The relative importance of DIT has recently been questioned.^10,11^ First, whilst a minimum of 2 T2 lesions are required for DIS (provided the lesions are in different MS-typical regions),^5^ many patients have a higher number of lesions at presentation.^12,13^ A higher number of T2 brain lesions, and greater fulfilment of the Barkhof criteria (reflecting both lesion number and location),^14^ is associated with a higher risk of a second clinical attack.^13^ Contrast-enhanced MRI is required to determine whether T2 lesions are of different ages^15^; however, some experts argue that in patients with a high burden of T2 lesions, the likelihood that all lesions formed simultaneously is very low.^10^ A high number of lesions in different regions may provide indirect evidence for DIT, although direct evidence is lacking.^10^ Second, the inclusion of CSF-specific OCBs in relapsing MS diagnostic criteria means that clinical/MRI evidence of DIT is no longer mandatory for MS diagnosis.^16^ Finally, with early use of high-efficacy disease-modifying therapies (DMTs), many patients have no evidence of inflammatory disease activity (relapses, new T2 lesions, gadolinium-enhancing lesions)^17^ and may not develop DIT in the short to medium term.

The aim of this study was to explore whether DIT (or positive CSF) is necessary to make a diagnosis of MS in patients with fulfilment of a high number of DIS criteria (lesions in ≥3 regions typically affected in MS, including the optic nerve). In patients with a typical first demyelinating event, we investigated the performance of DIS criteria alone for a diagnosis of MS, without DIT on MRI or CSF-specific OCBs.

## Methods

### Standard Protocol Approvals, Registrations, and Patient Consents

Patients provided written informed consent at the time of study entry and at follow-up. The study was approved by the institutional ethics committee.

### Participants

We studied patients with a first demyelinating event recruited from Moorfields Eye Hospital and the National Hospital for Neurology and Neurosurgery, London, the United Kingdom, as part of 2 prospective cohorts. All patients were older than 16 years and presented with a typical syndrome suggestive of MS (unilateral optic neuritis, partial myelitis, brainstem/cerebellar, or supratentorial syndrome). Alternative diagnoses were actively excluded by the treating neurologist/neuro-ophthalmologist with diagnostic testing available at the time of initial evaluation.

The first cohort were recruited between 1995 and 2004. Patients underwent MRI scans with proton density (PD)/T2-weighted and postcontrast T1-weighted scans of the brain and spinal cord within 3 months of onset on the same 1.5T Signa scanner. A subgroup of patients had lumbar puncture (LP) and/or visual evoked potentials (VEPs) as part of routine clinical care. The patients had scheduled follow-up at 3 months, 1, 3, and 5 years with clinical assessment (including collecting data on any relapses) and follow-up PD/T2-weighted MRI scans to look for new T2 lesions (and/or gadolinium-enhancing lesions on postcontrast T1-weighted scans). The cohort had a further follow-up after ∼15 years including MRI of the brain with 2D fluid-attenuated inversion recovery (FLAIR) sequences for lesion detection using the same 3T Philips Achieva TX scanner.

The second cohort were recruited between 2014 and 2021. All patients underwent MRI including 3D-FLAIR and precontrast and postcontrast T1-weighted scans of the brain, PD/T2-weighted sequences plus precontrast and postcontrast T1-weighted scans of the spinal cord, and 2D-coronal short tau inversion recovery sequences plus precontrast and postcontrast T1-weighted scans with fat saturation of the orbits. A subgroup of patients had LP performed as part of routine clinical care. The patients had scheduled clinical and MRI follow-up after 6 months, 1, 3, and 5 years to look for new T2 lesions on 3D-FLAIR in the brain and PD/T2-weighted sequences in the whole spinal cord (postcontrast T1-weighted imaging and orbital MRI was not performed at follow-up). Full details of MRI acquisition protocols for both cohorts have been described in detail previously.^8,12^

From each cohort, we selected patients with at least 1 follow-up MRI to look for DIT and >3 years of clinical follow-up or a diagnosis of MS using the 2017 McDonald criteria, whichever occurred first. Patients included in this analysis were not selected based on MRI findings, and we included patients with and without DIS and/or DIT at baseline.

### MRI Analysis and Other Paraclinical Testing

MRI scans were reviewed by experienced neuroradiologists blinded to the patients' clinical status. The number of T2-hyperintense and gadolinium-enhancing lesions and their location (periventricular, cortical/juxtacortical, infratentorial, spinal cord, optic nerve) was recorded. The number of new T2-hyperintense lesions at follow-up was also noted. CSF analysis was conducted using isoelectric focusing combined with immunoblotting. When ≥2 OCBs were present only in the CSF, they were considered to be CSF-specific. Full field pattern reversal VEPs were reported according to local normative data by a neurophysiologist.

### Application of Diagnostic Criteria to Baseline MRI Scans

We applied 3 DIS criteria to baseline MRI scans requiring lesions in at least 2 of 4 (≥2/4) regions typically affected in MS (i.e., 2017 McDonald DIS criteria), ≥3 of 4 (≥3/4) regions, and 4 of 4 (4/4) regions. In the subgroup of patients with optic nerve evaluation using orbital MRI or VEPs, we expanded the number of DIS regions from 4 to 5 to include optic nerve lesions. We then applied 4 DIS criteria requiring lesions in ≥2/5 regions, ≥3/5 regions, ≥4/5 regions, and 5/5 regions, including the optic nerve.

For reference, we also applied the 2017 McDonald criteria to MRI scans obtained at baseline requiring MRI evidence of DIS (T2 lesions in 2/4 regions) plus DIT (gadolinium-enhancing and nonenhancing lesions at baseline and/or CSF-specific OCBs).

### Statistical Analysis

Descriptive statistics were used to describe continuous variables as mean (SD) and categorical variables as percentages.

The performance of each DIS criteria (without DIT/positive CSF) was evaluated by calculating the sensitivity, specificity, accuracy, positive predictive value (PPV), and negative predictive value (NPV), with 95% CIs, for a diagnosis of MS using the 2017 McDonald criteria during follow-up. We also investigated the performance of each of the DIS criteria for a second clinical attack (i.e., clinically definite MS) and new T2 lesion(s) during follow-up. Given differences in the MRI acquisition protocols and field strengths between the 2 cohorts studied, the performance of the DIS criteria was tested separately in each cohort. We analyzed all patients first then patients with and without optic neuritis separately. Because time influences the performance of MS diagnostic criteria, we repeated all analyses in patients with >10 years of clinical follow-up.

All statistical analyses were performed using SPSS version 21 (IBM Corp., Armonk, NY).

### Data Availability

The corresponding author has full access to all the data in the study and takes responsibility for the data integrity and data analysis. The anonymized dataset is available from the corresponding author on reasonable request.

## Results

### Patient Characteristics

We included 244 patients with a first demyelinating event from a total of 262 patients (178 in the first cohort and 84 patients in the second cohort). Two patients from the first cohort developed alternative diagnoses during follow-up (1 each with neuromyelitis optica spectrum disorder [NMOSD] and myelin oligodendrocyte glycoprotein antibody-associated disorder [MOGAD]) and were excluded. The remaining 16 patients either had no follow-up MRI available to demonstrate DIT, <3 years of clinical follow-up, or both.

Approximately 80% of patients initially presented with optic neuritis, other demographic and clinical characteristics were typical of early relapsing MS (Table 1 and eTable 1). Patients were evaluated with brain and spinal cord MRI within 3 months of onset; 112 (46%) patients had optic nerve evaluation with either orbital MRI (n = 70) or VEPs (n = 42), and 58 (24%) patients underwent LP (Table 1).

**Table 1:** Demographic and Clinical Characteristics of Patients Included in the Study

	All patients (n = 244)	Patients with optic nerve evaluation (n = 112)
Age, y, mean (SD)	32.5 (7.4)	32.5 (7.6)
Female, n (%)	154 (63)	68 (61)
Optic neuritis, n (%)	197 (81)	79 (71)
Non-White ethnicity, n (%)^a^	50/229 (22)	21/98 (21)
Abnormal brain MRI, n (%)	202 (83)	100 (89)
Abnormal spinal MRI, n (%)	93 (38)	58 (52)
Abnormal optic nerve evaluation, n (%)^b^	—	89 (79)
Dissemination in space, n (%)		
0 region	41 (17)	1 (1)
1 region	43 (17)	11 (10)
2 regions	51 (21)	18 (16)
3 regions	61 (25)	25 (22)
4 regions	48 (20)	29 (26)
5 regions	—	28 (25)
Dissemination in time at baseline, n (%)	80 (33)	36 (32)
CSF-specific oligoclonal bands, n (%)^a^	45/58 (78)	25/31 (81)
Multiple sclerosis at last follow-up, n (%)	187 (77)	94 (77)
Second clinical attack, n (%)	119 (49)	45 (40)
New T2 lesion(s), n (%)	170 (70)	79 (71)

a Over the number of patients with data available.

b Optic nerve evaluation included visual evoked potentials (n = 42) or MRI (n = 70).

The baseline brain and spinal cord MRI was abnormal in 202 (83%) and 93 (38%) patients, respectively. Using the baseline brain and spinal cord MRI findings, 160 (66%) patients had lesions in ≥2/4 regions typically affected in MS, 109 patients had lesions in ≥3/4 (45%) regions, and 48 (20%) patients had lesions in 4/4 regions. When the 2 patient cohorts were analyzed separately, the second cohort had a higher proportion of patients with lesions in ≥3/4 regions compared with the first cohort (60% vs 39%) (eTable 1). In total, 104 (43%) patients had DIT on MRI and/or positive CSF at baseline.

Among patients who had optic nerve evaluation (n = 112), the demographic profile was similar to the whole cohort, but there were a higher proportion of patients with a nonoptic neuritis presentation (29% vs 19%) (Table 1). Eighty-nine (79%) patients had optic nerve lesions; 28/42 (67%) patients had abnormal VEPs (24/25 [96%] with optic neuritis, 4/17 [24%] with nonoptic neuritis presentations) in the first cohort, and 61/70 (87%) patients had abnormal orbital MRI in the second cohort. When the optic nerve was added as a fifth region, 100 (89%) patients had lesions in ≥2/5 regions, 82 (73%) patients had lesions in ≥3/5 regions, 57 (51%) patients had lesions in ≥4/5 regions, and 28 (25%) patients had lesions in 5/5 regions.

Over a mean follow-up of 11.2 (SD 9.3) years, 187 (77%) patients were diagnosed with MS using the 2017 McDonald criteria, 119 (49%) patients had a second clinical attack, and 170 (70%) patients had a new T2 lesion(s) at follow-up (Table 1). The median number of follow-up MRI scans was 4 (range 1–5) in the first cohort and 3 (range 1–4) for the second cohort. Among the 57 (23%) patients who did not develop MS, 52 (91%) had either normal MRI or involvement of a single CNS region, including 16 patients with optic neuritis who were evaluated with either VEPs (n = 6) or orbital MRI (n = 10). After a mean follow-up of 13.3 (SD 5.2) years in this group, the diagnosis remained “clinically isolated syndrome.”

Thirty-nine (16%) patients received treatment with DMTs before a second clinical attack, all of whom satisfied the 2017 McDonald criteria for a diagnosis of MS before starting treatment.

### Performance of the DIS Criteria

The performance of the various DIS criteria for a diagnosis of MS using the 2017 McDonald criteria, a second clinical attack or new T2 lesions(s) at follow-up is shown in Tables 2 and 3.

**Table 2:** Performance of DIS Criteria With Increasing Number of Regions in the Whole Cohort (n = 244)

	Sensitivity % (95% CI)	Specificity % (95% CI)	Accuracy % (95% CI)	PPV % (95% CI)	NPV % (95% CI)
Diagnosis of McDonald MS					
DIS alone					
DIS ≥2/4 regions	84.0 (77.9–88.9)	91.2 (80.7–97.1)	86.7 (80.6–89.8)	96.9 (93.1–98.6)	63.4 (55.3–70.8)
DIS ≥3/4 regions	57.8 (50.3–64.9)	98.2 (90.6–99.7)	67.2 (60.9–73.1)	99.1 (93.9–99.9)	41.5 (37.4–45.7)
DIS 4/4 regions	25.7 (19.6–32.6)	100 (93.7–100)	43.0 (36.7–49.5)	100 (92.6–100)	29.1 (27.4–30.9)
DIS + DIT at onset^a^	50.8 (43.1–58.0)	100 (94.2–100)	62.3 (56.7–69.2)	100 (96.1–100)	37.5 (37.3–44.4)
Second clinical attack					
DIS alone					
DIS ≥2/4 regions	81.5 (73.4–88.0)	48.0 (39.0–57.1)	64.3 (58.0–70.4)	59.9 (55.3–64.3)	73.2 (64.2–80.6)
DIS ≥3/4 regions	53.8 (44.4–63.0)	64.0 (55.0–72.4)	59.0 (52.6–65.3)	58.7 (51.6–65.5)	59.3 (53.5–64.8)
DIS 4/4 regions	24.4 (17.0–33.1)	84.8 (77.3–90.6)	55.3 (48.9–61.7)	60.4 (47.5–72.0)	54.1 (50.9–57.2)
DIS + DIT at onset^a^	52.9 (43.6–62.2)	76.8 (68.4–83.9)	65.2 (58.8–71.1)	68.5 (60.2–75.7)	63.2 (58.1–68.0)
New T2 lesion at follow-up					
DIS alone					
DIS ≥2/4 regions	82.4 (75.8,87.8)	70.3 (50.7,72.3)	78.7 (69.8,80.7)	81.4 (76.7,85.3)	63.4 (54.6,71.4)
DIS ≥3/4 regions	56.5 (48.7–64.1)	82.4 (71.8–90.3)	64.3 (58.0–70.4)	88.1 (81.6–92.5)	45.2 (40.3–50.2)
DIS 4/4 regions	26.5 (20.0–33.8)	95.9 (88.6–99.2)	47.5 (41.1–54.0)	93.8 (82.8–97.9)	36.2 (33.9–38.6)
DIS + DIT at onset^a^	47.6 (39.9–55.4)	85.1 (75.0–92.3)	59.0 (52.6–65.3)	88.0 (80.7–92.9)	41.4 (37.3–45.7)

Abbreviations: DIS = dissemination in space; DIT = dissemination in time; MS = multiple sclerosis; NPV = negative predictive value; PPV = positive predictive value.

a CSF-specific oligoclonal bands were considered as a substitute for MRI evidence of DIT at baseline.

**Table 3:** Performance of DIS Criteria With Increasing Number of Regions in the Subgroup of Patients With Optic Nerve Evaluation (n = 112)

	Sensitivity % (95% CI)	Specificity % (95% CI)	Accuracy % (95% CI)	PPV % (95% CI)	NPV % (95% CI)
Diagnosis of McDonald MS					
DIS alone					
DIS ≥2/5 regions	95.7 (89.5–98.8)	44.4 (21.5–69.2)	87.5 (79.9–93.0)	90.0 (85.6–93.2)	66.7 (40.2–85.6)
DIS ≥3/5 regions	83.0 (73.8–90.0)	83.3 (58.6–96.4)	83.0 (74.8–89.5)	95.1 (90.2–98.7)	50.0 (36.4–60.5)
DIS ≥4/5 regions	60.6 (50.0–70.6)	100 (81.5–100)	67.0 (57.4–75.6)	100 (93.7–100)	32.7 (27.5–38.5)
DIS 5/5 regions	29.8 (20.8–40.1)	100 (81.5–100)	41.1 (31.9–50.8)	100 (87.7–100)	21.4 (19.3–23.8)
DIS + DIT at onset^a^	53.9 (43.0–64.6)	100 (85.2–100)	63.4 (53.8–72.3)	100 (92.6–100)	35.9 (30.9–41.2)
Second clinical attack					
DIS alone					
DIS ≥2/5 regions	95.6 (84.9–99.5)	14.9 (7.4–25.7)	47.3 (37.8–57.0)	43.0 (40.1–45.9)	83.3 (53.5–96.6)
DIS ≥3/5 regions	80.0 (65.4–90.4)	31.3 (20.6–43.8)	50.9 (41.3–60.5)	43.9 (38.6–49.3)	70.0 (54.1–82.2)
DIS ≥4/5 regions	68.9 (52.4–81.8)	61.2 (48.5–72.9)	64.3 (54.7–73.1)	54.4 (45.4–63.1)	74.5 (64.6–82.5)
DIS 5/5 regions	35.6 (21.9–51.2)	82.1 (70.8–90.4)	63.4 (53.8–72.3)	57.1 (41.1–71.8)	65.5 (59.8–70.8)
DIS + DIT at onset^a^	57.8 (42.2–72.3)	67.2 (54.6–78.2)	63.4 (53.8–72.3)	54.2 (43.6–64.4)	70.3 (61.8–77.6)
New T2 lesion at follow-up					
DIS alone					
DIS ≥2/5 regions	94.9 (87.5–98.6)	24.2 (11.1–42.3)	74.1 (65.0–81.2)	75.0 (71.1–78.6)	66.7 (39.3–86.1)
DIS ≥3/5 regions	83.5 (73.5–90.9)	51.5 (33.5–69.2)	74.1 (65.0–81.9)	80.5 (74.1–85.6)	56.7 (41.9–70.4)
DIS ≥4/5 regions	63.3 (51.7–73.9)	78.8 (61.2–91.0)	67.9 (58.4–76.4)	87.7 (78.4–93.4)	47.3 (39.0–55.7)
DIS 5/5 regions	31.6 (21.6–43.1)	90.9 (75.7–98.1)	49.1 (39.5–58.7)	100 (73.0–100)	35.7 (31.6–40.1)
DIS + DIT at onset^a^	50.6 (39.1–62.1)	75.8 (57.7–88.9)	58.0 (48.3–67.3)	83.3 (72.5–90.5)	39.1 (32.3–46.3)

Abbreviations: DIS = dissemination in space; DIT = dissemination in time; MS = multiple sclerosis; NPV = negative predictive value; PPV = positive predictive value.

a CSF-specific oligoclonal bands were considered as a substitute for MRI evidence of DIT at baseline.

#### All Patients

In the whole cohort, DIS criteria requiring lesions in an increasing number of regions resulted in sensitivity of 84% for ≥2/4 regions falling to 58% for ≥3/4 regions and 26% for 4/4 regions for a diagnosis of MS using the 2017 McDonald criteria. By contrast, specificity for a diagnosis of McDonald MS increased from 91% for ≥2/4 regions to 98% for ≥3/4 regions and was 100% for 4/4 regions. The PPV of ≥3/4 and 4/4 regions were both very high (≥99%).

The findings were similar for development of a second clinical attack or new T2 lesion(s) during follow-up with moderate to low sensitivity, but moderate to very high specificity with fulfilment of a greater number of DIS criteria (Table 2).

When comparing the specificity for each of the DIS criteria for a second clinical attack vs a diagnosis of MS using the McDonald criteria, the specificity was generally lower for the development of a second attack, driven by the low number of patients with relapses (Table 2).

For reference, we also applied the 2017 McDonald DIS and DIT criteria to baseline MRI scans (with CSF-specific OCBs as a substitute for MRI evidence of DIT, where available). The diagnostic performance was similar to the more stringent DIS criteria requiring ≥3/4 regions (Table 2).

#### Subgroup of Patients With Optic Nerve Evaluation

The analyses were repeated in patients with optic nerve evaluation to expand the number of DIS regions from 4 to 5 (Table 3). DIS criteria requiring lesions in a higher number of regions exhibited sensitivities ranging from 83% for ≥3/5 regions to 30% for 5/5 regions for an MS diagnosis using the 2017 McDonald criteria. The specificity of lesions in ≥3 regions for a diagnosis of 2017 McDonald MS was moderate to very high (83% for ≥3/5 regions, 100% for ≥4/5 regions, and 100% for 5/5 regions). The PPV of ≥3/5, ≥4/5, or 5/5 regions for MS diagnosis was also very high (>95%).

For reference, the 2017 McDonald DIS and DIT criteria applied to baseline MRI scans (and/or CSF examination) exhibited similar sensitivity (54% vs 61%) and specificity (100% for both) compared with the modified DIS criteria requiring lesions in ≥4/5 regions.

Using the outcome of a second clinical attack or new T2 lesion(s) at follow-up, the performances of the various DIS criteria was similar in patients with optic nerve evaluation. DIS criteria requiring lesions in a higher number of regions resulted in falling sensitivity but increasing specificity and PPV for a second clinical attack or DIT on follow-up MRI.

#### Analysis of Each Cohort Separately

DIS criteria requiring a higher number of regions resulted in increasing specificity and PPV for a diagnosis of 2017 McDonald MS but falling sensitivity (eTables 2 and 3). In the cohort who underwent MRI at 3T with an optimized protocol for lesion detection including 3D-FLAIR, the sensitivity of DIS requiring ≥3/4 (70 vs 52%) or 4/4 (32 vs 23%) regions was higher compared with patients in the first cohort (eTable 2). The findings were similar for the secondary outcomes of CDMS and new T2 lesions at follow-up (data not shown).

### Performance of the DIS Criteria in Patients With and Without Optic Neuritis

The performance of the DIS criteria for a diagnosis of MS using the 2017 McDonald criteria in patients with optic neuritis and nonoptic neuritis presentations is presented in Table 4. The findings were similar with involvement of ≥4 regions in DIS displaying high specificity and PPV for a diagnosis of 2017 McDonald MS in patients with and without optic neuritis. In the subgroup of patients with optic nerve evaluation, the sensitivity of ≥4/5 regions in DIS was lower among patients with a nonoptic neuritis presentation compared with the optic neuritis patients (47 vs 68%), but similar to the sensitivity of the 2017 McDonald DIS and DIT criteria when applied baseline MRI scans and CSF findings (47% for both). The findings were similar when considering a second clinical attack or new T2 lesions at follow-up to the whole cohort with falling sensitivity with lesions in a greater number of DIS regions but increasing specificity (eTables 4 and 5).

**Table 4:** Performance of DIS Criteria in Patients With and Without Optic Neuritis for a Diagnosis of 2017 McDonald Multiple Sclerosis

	Sensitivity % (95% CI)	Specificity % (95% CI)	Accuracy % (95% CI)	PPV % (95% CI)	NPV % (95% CI)	Sensitivity % (95% CI)	Specificity % (95% CI)	Accuracy % (95% CI)	PPV % (95% CI)	NPV % (95% CI)
Diagnosis of McDonald MS	Optic neuritis (n = 197)					Nonoptic neuritis (n = 47)				
DIS alone										
DIS ≥2/4 regions	82.2 (75.2–88.0)	88.9 (76.0–96.3)	83.8 (77.8–88.6)	96.2 (91.6–98.3)	83.8 (77.9–88.6)	88.6 (72.3–96.8)	91.7 (61.5–99.8)	89.4 (76.9–96.5)	96.9 (82.5–99.5)	73.3 (51.8–87.5)
DIS ≥3/4 regions	54.0 (45.7–62.1)	97.8 (88.2–99.9)	64.0 (56.8–70.1)	98.8 (92.5–99.8)	38.6 (34.5–42.9)	74.3 (56.7–87.5)	100 (73.5–100)	80.9 (66.7–90.9)	100 (86.8–100)	57.1 (43.2–70.1)
DIS 4/4 regions	23.0 (16.6–30.5)	100 (92.1–100)	40.6 (33.7–47.8)	100 (90.0–100)	27.8 (26.1–30.0)	37.1 (21.5–55.1)	100 (73.5–100)	53.2 (38.1–67.9)	100 (75.3–100)	35.3 (29.7–41.3)
DIS + DIT at onset^a^	46.1 (38.0–54.3)	100 (92.1–100)	58.4 (51.2–65.3)	100 (94.9–100)	35.4 (32.2–38.9)	62.9 (44.9–78.5)	100 (73.5–100)	72.3 (57.4–84.4)	100 (84.6–100)	48.0 (37.5–58.7)
Diagnosis of McDonald MS in patients with optic nerve evaluation	Optic neuritis (n = 79)					Nonoptic neuritis (n = 33)				
DIS alone										
DIS ≥2/5 regions	96.8 (89.0–99.6)	37.5 (15.2–64.6)	84.8 (75.0–91.9)	85.9 (80.6–89.9)	75.0 (40.0–93.1)	93.3 (77.9–99.2)	66.7 (9.4–99.2)	90.9 (75.7–98.1)	96.6 (84.9–99.3)	50.0 (17.4–82.6)
DIS ≥3/5 regions	82.5 (70.9–91.0)	75 (47.6–92.7)	81.0 (70.6–89.0)	92.9 (84.7–96.8)	52.2 (37.3–66.7)	86.7 (69.3–96.2)	100 (29.2–100)	87.9 (71.8–96.6)	100 (86.8–100)	42.9 (23.2–65.1)
DIS ≥4/5 regions	68.3 (55.3–79.4)	100 (79.4–100)	74.7 (63.6–83.8)	100 (91.8–100)	44.4 (35.8–53.5)	46.7 (28.3–65.7)	100 (29.2–100)	51.5 (33.5–69.2)	100 (76.8–100)	15.8 (11.8–20.8)
DIS 5/5 regions	33.3 (22.0–46.3)	100 (79.4–100)	46.8 (33.5–58.4)	100 (84.0–100)	27.6 (24.2–31.2)	23.3 (9.9–42.3)	100 (29.2–100)	30.3 (15.6–48.7)	100 (59.0–100)	11.5 (9.7–13.7)
DIS + DIT at onset^a^	46.0 (33.4–59.1)	100 (78.4–100)	57.0 (45.3–68.1)	100 (88.1–100)	32.0 (27.3–83.2)	46.7 (28.3–65.7)	100 (29.2–100)	51.5 (33.5–69.2)	100 (76.8–100)	15.8 (11.8–20.8)

Abbreviations: DIS = dissemination in space; DIT = dissemination in time; MS = multiple sclerosis; NPV = negative predictive value; PPV = positive predictive value.

a CSF-specific oligoclonal bands were considered as a substitute for MRI evidence of DIT at baseline.

### Performance of the DIS Criteria in Patients With Long-Term Follow-Up

A total of 163 (67%) patients had >10 years of follow-up. In this group, 118 (72%) patients were diagnosed with MS using the 2017 McDonald criteria, 98 (60%) patients had a second clinical attack, and 112 (69%) patients had a new T2 lesion on a follow-up MRI. In the patients with optic nerve evaluation, 39 patients had >10 years of follow-up and outcomes were similar; 31 (79%) patients were diagnosed with MS, 26 (67%) patients had a second clinical attack, and 27 (69%) patients had new T2 lesion(s) at follow-up.

The performances of the various DIS criteria for the diagnosis of MS using the 2017 McDonald criteria are shown in eTable 6. In all patients with >10 years of follow-up, the sensitivity, specificity, and PPV for a diagnosis of McDonald MS were similar to the whole cohort, but the specificity for a second clinical attack was higher in the longer term (80% for ≥3/4 regions and 94% for 4/4 regions). In the subgroup of patients with optic nerve evaluation, the specificity for both a diagnosis of MS using the McDonald criteria (100% for ≥4/5 regions, 100% for 5/5 regions) or a second clinical attack (92% for ≥4/5 regions, 100% for 5/5 regions) was very high. The findings were similar for development of a second clinical attack or new T2 lesion(s) on MRI. Full results are presented in eTable 7.

### Algorithm for MS Diagnosis Based on Fulfilment of DIS

Taking into account our findings, we developed an algorithm for MS diagnosis based on DIS criteria fulfilment (Figure). In patients with a first demyelinating event, a diagnosis of MS can be made if there are brain and spinal cord lesions on MRI in 4/4 typical regions, and DIT on MRI or positive CSF are not mandatory. If this criterion is not fulfilled, optic nerve evaluation should be considered, so that DIS in up to 5 regions can be evaluated. In patients with lesions in 4/5 typical regions, including the optic nerve, MS can also be diagnosed without DIT/positive CSF. Based on our findings 25%–40% of people with a first demyelinating event may diagnosed with MS, after other disorders have been excluded, based on high fulfilment of DIS criteria in ≥4/5 typical regions. In the remaining patients, particularly those with DIS in 2–3 regions after optic nerve evaluation, additional evidence is required to make a diagnosis of MS, either DIT on MRI, positive CSF, or both.

**Figure:**
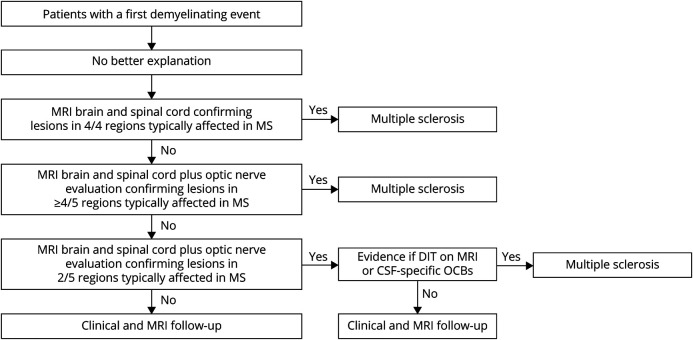
Proposed Algorithm for MS Diagnosis in Patients With a First Demyelinating Event DIT = dissemination in time; MS = multiple sclerosis; OCB = oligoclonal band.

## Discussion

We evaluated the performance of high fulfilment of DIS criteria for MS diagnosis, a second clinical attack, or new T2 lesion(s) on MRI, in patients with a first demyelinating event over ∼11 years. DIS criteria requiring lesions on MRI in 4/4 MS typical regions displayed 100% specificity and PPV for each of the outcomes tested, but moderate to low sensitivity and moderate accuracy. Notably, DIS criteria requiring lesions in 4/4 regions had similar specificity and PPV as the 2017 McDonald criteria requiring both DIS and DIT/positive CSF combined, when applied to baseline MRI scans. These findings suggest that DIS alone in 4/4 regions seems sufficient to “rule-in” MS, without the need for DIT or positive CSF, with a negligible risk of a false-positive MS diagnosis. However, the sensitivity of DIS in 4/4 regions was relatively low, especially in patients scanned at lower field strengths and imaging protocols not optimized to detect MS lesions. Lesser fulfilment of DIS criteria does not “rule-out” MS, and CSF examination and/or follow-up MRI should be considered in this patient group.

Recent studies have demonstrated the utility of including optic nerve lesions in DIS criteria.^6,7,18,19^ In patients with optic nerve evaluation, the findings were broadly similar with increasing specificity of DIS criteria requiring lesions in 3, 4, or 5 regions but reducing sensitivity. Notably, the specificity and PPV of 4/5 or 5/5 regions was high and similar to the 2017 McDonald criteria requiring both DIS and DIT/positive CSF at baseline. A lower threshold for DIS in 3/5 regions had higher sensitivity, but the specificity was lower. A reduction in specificity when optic nerve lesions are included in DIS criteria has been noted previously.^7,19^ Studies in larger cohorts of patients with complete evaluation of optic nerve are needed to determine whether a lower threshold of 3/5 regions would be more appropriate, accepting that the performance of MS diagnostic criteria requires a trade-off between sensitivity and specificity.

Based on our findings we propose an algorithm whereby MS can be diagnosed on the basis of DIS alone without DIT/positive CSF in some patients (Figure). Using the algorithm, if a patient has a typical MS presentation and an MRI shows high fulfilment of DIS criteria, then the diagnosis could be established and treatment started without the need for further testing. A diagnosis of MS could be made in patients at very high risk intuitively (lesions in ≥4 typical regions) who have no gadolinium enhancement (>70% of patients with a recent first demyelinating event^15^) who are unable or unwilling to undergo LP, or if LP might be delayed, which is common in some health care settings, particularly outside specialist centers. Using DIS alone could simplify the workup of patients with suspected MS. Contrast-enhanced MRI scans require venous cannulation and staff to administer contrast agents, plus increased scanning times to acquire precontrast and postcontrast T1-weighted images, or repeat MRI scanning if the initial diagnostic MRI was performed without contrast. In general, there is a move to limit use of gadolinium because of the uncertain clinical significance of deposition in brain tissue^20^ and the impact on the aquatic environment.^21^ The use of CSF-specific OCBs to provide a substitute for clinical or MRI evidence of DIT results in earlier MS diagnosis^16^; however, compared with MRI scans, LP is invasive, requires greater healthcare resources, and is less acceptable to patients undergoing MS workup.^22^ Complications of LP such as postdural puncture headache sometimes necessitate emergency department visits or additional treatment such as epidural blood patch.^23^ CSF-specific OCBs are found in approximately 90% of patients with established MS but only 70% first demyelinating event.^24^ Although considered a potential “red flag,”^25^ the differential diagnosis is rather limited in a patient with a typical MS presentation, MRI evidence of DIS in 4–5 typical regions but negative OCBs. The proposed diagnostic algorithm may reduce health care costs, particularly in low-income countries where the cost of investigations and a lack of specialist laboratory equipment represent significant barriers.^26^ Despite these limitations, as set out in our diagnostic algorithm, both contrast-enhanced MRI and LP will remain essential tools in the workup of patients with suspected MS, certainly in patients with lesser fulfilment of DIS criteria but also to aid in differential diagnosis^25^ and potentially in counselling patients about prognosis.^12,13^

Removing the requirement for DIT/positive CSF from MS diagnostic criteria in selected patients with high DIS fulfilment comes with some potential concerns. The combination of both DIS and DIT improves MS diagnostic criteria performance by enhancing specificity.^6,19^ Previous studies have typically applied less stringent DIS criteria requiring involvement of only 2 regions to confirm DIS,^6,19^ whereas our findings suggest that a higher bar for DIS fulfilment maintains high specificity without the need for DIT. Misdiagnosis of MS in patients with nonspecific or vascular white matter lesions represents an ongoing challenge posed by MRI-based diagnostic criteria.^27^ Surveys in North America, where the rate of MS misdiagnosis appears to be high,^28^ suggest that misunderstanding of MS diagnostic criteria is a significant contributor to misdiagnosis.^29,30^ Application of DIS criteria requires careful evaluation of lesions to differentiate periventricular and juxtacortical in particular from nonspecific subcortical lesions.^5,29^ Educational efforts to increase the knowledge and confidence of neurologists evaluating patients with suspected MS^30^ and increased access to neuroradiologists and specialist MS care^31^ are priorities to reduce misdiagnosis. Accuracy of MS diagnosis may be enhanced by DIS criteria requiring lesions in a higher number of regions, including the optic nerve or spinal cord, which are not typically affected by common MS mimics, such as migraine or small vessel cerebrovascular disease. Antibody-mediated disorders such as NMOSD and MOGAD also frequently involve both the optic nerve and spinal cord and specific clinical and MRI findings typically aid in differential diagnosis.^25^

This study does have some limitations. First, patients with optic neuritis were over-represented. Some studies have found that patients with optic neuritis have a more favorable prognosis, including lower risk of a second clinical attack or new lesions on MRI.^13^ This would be expected to negatively influence specificity in this cohort, whereas we found consistently high specificity for DIS criteria requiring lesions in ≥4 typical regions. This may reflect the recruitment of optic neuritis patients from specialist neuro-ophthalmology clinics and the high pretest probability for MS with >80% of patients having abnormal brain MRI.^12,13^ We repeated analyses in patients with and without optic neuritis; the specificity and PPV of DIS criteria requiring lesions in ≥4 regions was the same. As expected, the sensitivity of criteria that included optic nerve lesions (DIS in ≥4/5 regions) was higher in patients with optic neuritis reflecting the low frequency of asymptomatic optic nerve lesions in patients with brainstem or spinal cord syndromes (<20% patients).^7^ Optical coherence tomography, which was not consistently available in this study, may aid in detection of asymptomatic optic nerve lesions.^7^ Second, not all patients underwent CSF examination. Most of the patients (>97%) who did not undergo LP already had either evidence of DIT on MRI (or a second clinical attack during follow-up), did not have MRI evidence of DIS, or both. The low rate of CSF examination is therefore unlikely to influence our findings. Third, we analyzed 2 cohorts recruited over a period of 3 decades. Although recruitment of the cohorts was very similar, reflected in the consistency of the demographic and clinical profile, there were differences in the MRI study protocols, including scanning at 3T in the second cohort, and use 3D FLAIR to improve lesion detection. While this heterogeneity is a limitation, it may better reflect routine clinical practice where patients with suspected MS are evaluated with MRI at different field strengths and with protocols not optimized for detection of demyelinating lesions.^32^ Fourth, susceptibility-weight imaging or T2*-weighted sequences were not part of the study protocols, and we were unable to evaluate for central vein sign (CVS) or paramagnetic rim lesions (PRLs). These imaging biomarkers have high specificity for MS^33,34^ but add additional scanning time to established MRI protocols and have not yet entered routine clinical practice. In patients with a first demyelinating event, conventional MRI alone demonstrating DIS in ≥4 typical regions may be sufficient to make a diagnosis of MS, although CVS or PRLs might still be useful in patients with DIS in ≤2 typical regions and in patients with diagnostic uncertainty or vascular comorbidities.^35,36^ The algorithm presented could be adapted if these imaging techniques become widely available. Finally, we evaluated young adult patients with typical clinical presentations suggestive of MS where alternative diagnoses had been actively excluded before study entry, which will influence specificity. The performance of more stringent DIS criteria alone without DIT/positive CSF should be tested in in patients with atypical presentations (including radiologically isolated syndrome), primary progressive MS, and special patient populations including children, older adults, and patients with comorbidities.

In conclusion, in patients with a first demyelinating event, lesions on MRI involving ≥4 regions typically affected in MS (periventricular, cortical/juxtacortical, infratentorial, spinal cord, optic nerve) has very high specificity and PPV for a diagnosis of MS, suggesting that false-positive results are unlikely. DIT/positive OCBs may not be necessary to confirm a diagnosis of MS in patients with typical clinical presentations and DIS in ≥4 regions, facilitating the application of the criteria in clinical practice and streamlining MS diagnosis.

## Glossary

CVS: central vein sign
DIS: dissemination in space
DIT: dissemination in time
DMT: disease-modifying therapy
FLAIR: fluid-attenuated inversion recovery
LP: lumbar puncture
MOGAD: myelin oligodendrocyte glycoprotein antibody-associated disorder
MS: multiple sclerosis
NMOSD: neuromyelitis optica spectrum disorder
NPV: negative predictive value
OCB: oligoclonal band
PD: proton density
PPV: positive predictive value
PRL: para-magnetic rim lesion
VEP: visual evoked potential

## References

[1] Schumacher GA, Beebe G, Kibler RF, et al. Problems of experimental trials of therapy in multiple sclerosis: report by the panel on the evaluation of experimental trials of therapy in multiple sclerosis. Ann NY Acad Sci. 1965;122:552-568. doi:10.1111/j.1749-6632.1965.tb20235.x 14313512

[2] Brownlee WJ, Hardy TA, Fazekas F, Miller DH. Diagnosis of multiple sclerosis: progress and challenges. Lancet. 2017;389(10076):1336-1346. doi:10.1016/S0140-6736(16)30959-X 27889190

[3] Brownlee WJ, Swanton JK, Altmann DR, Ciccarelli O, Miller DH. Earlier and more frequent diagnosis of multiple sclerosis using the McDonald criteria. J Neurol Neurosurg Psychiatry. 2015;86(5):584-585. doi:10.1136/jnnp-2014-308675 25412872 PMC4451169

[4] Tintore M, Cobo-Calvo A, Carbonell P, et al. Effect of changes in MS diagnostic criteria over 25 years on time to treatment and prognosis in patients with clinically isolated syndrome. Neurology. 2021;97(17):e1641-e1652. doi:10.1212/WNL.0000000000012726 34521693

[5] Thompson AJ, Banwell BL, Barkhof F, et al. Diagnosis of multiple sclerosis: 2017 revisions of the McDonald criteria. Lancet Neurol. 2018;17(2):162-173. doi:10.1016/S1474-4422(17)30470-2 29275977

[6] Brownlee WJ, Miszkiel KA, Tur C, Barkhof F, Miller DH, Ciccarelli O. Inclusion of optic nerve involvement in dissemination in space criteria for multiple sclerosis. Neurology. 2018;91(12):e1130-e1134. doi:10.1212/WNL.0000000000006207 30120132 PMC6161554

[7] Vidal-Jordana A, Rovira A, Calderon W, et al. Adding the optic nerve in multiple sclerosis diagnostic criteria: a longitudinal, prospective, multicenter study. Neurology. 2024;102(1):e200805. doi:10.1212/WNL.0000000000207805 38165378 PMC10834130

[8] Foster MA, Pontillo G, Davagnanam I, et al. Improving criteria for dissemination in space in multiple sclerosis by including additional regions. Ann Clin Transl Neurol. 2024;11(10):2572-2582. doi:10.1002/acn3.52170 39078773 PMC11514922

[9] Arrambide G, Tintore M, Espejo C, et al. The value of oligoclonal bands in the multiple sclerosis diagnostic criteria. Brain. 2018;141(4):1075-1084. doi:10.1093/brain/awy006 29462277

[10] Miller AE. Dissemination in time as a requirement for diagnosis of multiple sclerosis: time for a change? Mult Scler. 2024;30(4-5):479-482. doi:10.1177/13524585241233999 38411037

[11] Toosy AT, Barkhof F. Multiple sclerosis can be diagnosed solely with dissemination in space: yes. Mult Scler. 2024;30(6):637-639. doi:10.1177/13524585241245313 38616522 PMC11071589

[12] Brownlee WJ, Altmann DR, Prados F, et al. Early imaging predictors of long-term outcomes in relapse-onset multiple sclerosis. Brain. 2019;142(8):2276-2287. doi:10.1093/brain/awz156 31342055

[13] Tintore M, Rovira A, Rio J, et al. Defining high, medium and low impact prognostic factors for developing multiple sclerosis. Brain. 2015;138(pt 7):1863-1874. doi:10.1093/brain/awv105 25902415

[14] Barkhof F, Filippi M, Miller DH, et al. Comparison of MRI criteria at first presentation to predict conversion to clinically definite multiple sclerosis. Brain. 1997;120(pt 11):2059-2069. doi:10.1093/brain/120.11.2059 9397021

[15] Rovira A, Swanton J, Tintore M, et al. A single, early magnetic resonance imaging study in the diagnosis of multiple sclerosis. Arch Neurol. 2009;66(5):587-592. doi:10.1001/archneurol.2009.49 19433658

[16] van der Vuurst de Vries RM, Mescheriakova JY, Wong YYM, et al. Application of the 2017 revised McDonald criteria for multiple sclerosis to patients with a typical clinically isolated syndrome. JAMA Neurol. 2018;75(11):1392-1398. doi:10.1001/jamaneurol.2018.2160 30083703 PMC6248116

[17] Simonsen CS, Flemmen HO, Broch L, et al. Early high efficacy treatment in multiple sclerosis is the best predictor of future disease activity over 1 and 2 years in a Norwegian population-based registry. Front Neurol. 2021;12:693017. doi:10.3389/fneur.2021.693017 34220694 PMC8248666

[18] Vidal-Jordana A, Rovira A, Arrambide G, et al. Optic nerve topography in multiple sclerosis diagnosis: the utility of visual evoked potentials. Neurology. 2021;96(4):e482-e490. doi:10.1212/WNL.0000000000011339 33328323 PMC7905792

[19] Filippi M, Preziosa P, Meani A, et al. Prediction of a multiple sclerosis diagnosis in patients with clinically isolated syndrome using the 2016 MAGNIMS and 2010 McDonald criteria: a retrospective study. Lancet Neurol. 2018;17(2):133-142. doi:10.1016/S1474-4422(17)30469-6 29275979

[20] Starekova J, Pirasteh A, Reeder SB. Update on gadolinium based contrast agent safety, from the AJR special series on contrast media. AJR Am J Roentgenol. 2024;223(3):e2330036. doi:10.2214/AJR.23.30036 37850581

[21] Brunjes R, Hofmann T. Anthropogenic gadolinium in freshwater and drinking water systems. Water Res. 2020;182:115966. doi:10.1016/j.watres.2020.115966 32599421 PMC7256513

[22] Christopher A, Brandon L, Clare B, et al. Patients' experience of the multiple sclerosis diagnostic pathway. J Neurol Neurosurg Psychiatry. 2023;94:A65-A66.

[23] Uppal V, Russell R, Sondekoppam R, et al. Consensus practice guidelines on postdural puncture headache from a multisociety, international working group: a summary report. JAMA Netw Open. 2023;6(8):e2325387. doi:10.1001/jamanetworkopen.2023.25387 37581893

[24] Dobson R, Ramagopalan S, Davis A, Giovannoni G. Cerebrospinal fluid oligoclonal bands in multiple sclerosis and clinically isolated syndromes: a meta-analysis of prevalence, prognosis and effect of latitude. J Neurol Neurosurg Psychiatry. 2013;84(8):909-914. doi:10.1136/jnnp-2012-304695 23431079

[25] Solomon AJ, Arrambide G, Brownlee WJ, et al. Differential diagnosis of suspected multiple sclerosis: an updated consensus approach. Lancet Neurol. 2023;22(8):750-768. doi:10.1016/S1474-4422(23)00148-5 37479377

[26] Solomon AJ, Marrie RA, Viswanathan S, et al. Global barriers to the diagnosis of multiple sclerosis: data from the Multiple Sclerosis International Federation Atlas of MS, Third Edition. Neurology. 2023;101(6):e624-e635. doi:10.1212/WNL.0000000000207481 37321866 PMC10424832

[27] Solomon AJ, Bourdette DN, Cross AH, et al. The contemporary spectrum of multiple sclerosis misdiagnosis: a multicenter study. Neurology. 2016;87(13):1393-1399. doi:10.1212/WNL.0000000000003152 27581217 PMC5047038

[28] Kaisey M, Solomon AJ, Luu M, Giesser BS, Sicotte NL. Incidence of multiple sclerosis misdiagnosis in referrals to two academic centers. Mult Scler Relat Disord. 2019;30:51-56. doi:10.1016/j.msard.2019.01.048 30738280

[29] Solomon AJ, Pettigrew R, Naismith RT, Chahin S, Krieger S, Weinshenker B. Challenges in multiple sclerosis diagnosis: misunderstanding and misapplication of the McDonald criteria. Mult Scler. 2021;27(2):250-258. doi:10.1177/1352458520910496 32162581

[30] Solomon AJ, Kaisey M, Krieger SC, et al. Multiple sclerosis diagnosis: knowledge gaps and opportunities for educational intervention in neurologists in the United States. Mult Scler. 2022;28(8):1248-1256. doi:10.1177/13524585211048401 34612110 PMC9189717

[31] Brownlee WJ, Ciccarelli O. All relapsing multiple sclerosis patients should be managed at a specialist clinic: yes. Mult Scler. 2016;22(7):873-875. doi:10.1177/1352458516636474 27207459

[32] Fernandes L, Allen CM, Williams T, et al. The contemporary role of MRI in the monitoring and management of people with multiple sclerosis in the UK. Mult Scler Relat Disord. 2021;55:103190. doi:10.1016/j.msard.2021.103190 34365316

[33] Sinnecker T, Clarke MA, Meier D, et al. Evaluation of the central vein sign as a diagnostic imaging biomarker in multiple sclerosis. JAMA Neurol. 2019;76(12):1446-1456. doi:10.1001/jamaneurol.2019.2478 31424490 PMC6704746

[34] Meaton I, Altokhis A, Allen CM, et al. Paramagnetic rims are a promising diagnostic imaging biomarker in multiple sclerosis. Mult Scler. 2022;28(14):2212-2220. doi:10.1177/13524585221118677 36017870 PMC9679799

[35] Clarke MA, Samaraweera AP, Falah Y, et al. Single Test to ARrive at Multiple Sclerosis (STAR-MS) diagnosis: a prospective pilot study assessing the accuracy of the central vein sign in predicting multiple sclerosis in cases of diagnostic uncertainty. Mult Scler. 2020;26(4):433-441. doi:10.1177/1352458519882282 31668125

[36] Lapucci C, Tazza F, Rebella S, et al. Central vein sign and diffusion MRI differentiate microstructural features within white matter lesions of multiple sclerosis patients with comorbidities. Front Neurol. 2023;14:1084661. doi:10.3389/fneur.2023.1084661 36970546 PMC10030505

